# A diagnostic index for predicting heart rate variability decline and prognostic value in newly diagnosed non-small cell lung cancer patients

**DOI:** 10.3389/fonc.2024.1463805

**Published:** 2024-12-04

**Authors:** Lifang Zhang, Ying Liu, Di Han, Yan Wang, Fanqi Geng, Wei Ding, Xuejuan Zhang

**Affiliations:** ^1^ Department of General Medicine, The Affiliated Hospital of Qingdao University, Qingdao, China; ^2^ Department of Infectious Disease, The Affiliated Hospital of Qingdao University, Qingdao, China

**Keywords:** heart rate variability, non-small cell lung cancer, diagnostic index, resting heart rate, serum sodium, interleukin-6, overall survival

## Abstract

**Background:**

Heart rate variability (HRV) is an important marker of autonomic nervous system function and cardiovascular health. Holter monitoring is a crucial method for evaluating HRV, but the procedure and result analysis are relatively complex. This study aims to develop a simplified diagnostic index for predicting HRV decline in newly diagnosed non-small cell lung cancer (NSCLC) patients and evaluate its prognostic value.

**Methods:**

This retrospective cross-sectional study included 131 newly diagnosed NSCLC patients. Baseline characteristics were compared between normal HRV group and declined HRV group. Univariate and multivariate logistic regression analyses identified significant predictors of HRV decline. A diagnostic index was developed based on resting heart rate (RHR), serum sodium, and interleukin-6 (IL-6) and externally validated. Kaplan-Meier survival analysis assessed the prognostic value of the index.

**Results:**

Patients with declined HRV had higher median RHR (84 b.p.m. vs. 70 b.p.m., *p* < 0.001), lower serum sodium (136.3 mmol/L vs. 138.7 mmol/L, *p* < 0.001), lower serum albumin (39 g/L vs. 41 g/L, *p* = 0.031), higher lactate dehydrogenase (LDH) (202 U/L vs. 182 U/L, *p* = 0.010), and higher IL-6 (11.42 pg/ml vs. 5.67 pg/ml, *p* < 0.001). Multivariate analysis identified RHR (OR = 3.143, *p* = 0.034), serum sodium (OR = 6.806, *p* < 0.001), and IL-6 (OR = 3.203, *p* = 0.033) as independent predictors of HRV decline. The diagnostic index, with an area under the curve (AUC) of 0.849, effectively predicted HRV decline. ROC analysis of the external validation data demonstrated an AUC of 0.788. Survival analysis showed that patients with a diagnostic index > 2 had significantly worse overall survival (log-rank *p* < 0.001).

**Conclusions:**

The study identified key clinical parameters that predict HRV decline in newly diagnosed NSCLC patients. The developed diagnostic index, based on RHR, serum sodium, and IL-6, effectively stratifies patients by HRV status and has significant prognostic value, aiding in early identification and management of high-risk patients.

## Introduction

Heart rate variability (HRV) serves as a significant indicator of autonomic nervous system function and cardiovascular health, reflecting the heart’s ability to adapt to various physiological and pathological conditions (1). Previous studies have confirmed that HRV is associated with the prognosis of cardiovascular disease, the occurrence of diabetic complications and mental illness (2–4). In oncology, autonomic dysfunction marked by decreased HRV can signal a higher risk of cardiovascular events and overall mortality (5). Holter monitoring is a crucial method for evaluating HRV (6). However, the procedure and result analysis are relatively complex, making it particularly important to find relatively simple and objective evaluation indicators.

Non-small cell lung cancer (NSCLC) constitutes about 85% of all lung cancer cases, making it the most prevalent type of lung cancer globally (7). Despite therapeutic advancements, the prognosis for NSCLC remains grim, with a five-year survival rate around 15% (8). Identifying reliable prognostic markers is essential for enhancing patient outcomes. Previous studies have suggested that HRV may serve as a non-invasive and easily measurable prognostic marker in cancer patients (9–11), but the current literature lacks a specific diagnostic index that can predict HRV decline and its impact on survival prognosis in NSCLC patients.

This study aims to develop a diagnostic index based on resting heart rate (RHR), serum sodium, and interleukin-6 (IL-6) levels to predict HRV decline in newly diagnosed NSCLC patients. By analyzing the relationship between these clinical parameters and HRV, we intend to establish a reliable predictive tool for early identification of high-risk patients. Compared to traditional HRV monitoring methods, such an index could offer a simplified assessment approach, significantly enhance personalized treatment strategies and potentially improving survival outcomes for patients with this challenging diagnosis.

## Methods

### Participants

This study is a retrospective observational cross-sectional study. 131 patients with newly diagnosed NSCLC in the Affiliated Hospital of Qingdao University from January 2018 to December 2023 were analyzed, followed by external validation with 43 patients enrolled between January 2015 and December 2017. The inclusion criteria were(1) aged 18-80years; (2) newly diagnosed NSCLC confirmed by pathology; (3) 24-hour Holter monitoring performed before treatment. The exclusion criteria for patients were (1) heart diseases; (2) diabetes mellitus; (3) mental illness; (4) pacemaker implantation; (5) estimated glomerular filtration rate(eGFR) < 30 ml/min/1.73m^2^; (6) alanine aminotransferase > 120 U/L; (7) taking drugs that affect heart rate; (8) incomplete medical records or lost follow up.

This study was approved by the Ethical Review Committee of the Affiliated Hospital of Qingdao University and performed in accordance with the Declaration of Helsinki. All patients signed informed consent and agreed to the relevant examination and treatment.

### Data collection

All relevant demographic characteristics, clinical features, and auxiliary examination results at the time of NSCLC diagnosis were collected from medical records. The tumor staging of NSCLC was based on American Joint Committee on Cancer(AJCC) Cancer Staging Manual (8th edition) (12). Laboratory parameters included hemoglobin, albumin (ALB), high-density lipoprotein cholesterol (HDL), low-density lipoprotein cholesterol (LDL), lactate dehydrogenase (LDH), serum creatinine, uric acid(UA), serum electrolytes (potassium, sodium, calcium) and IL-6. The eGFR was assessed using the Chronic Kidney Disease Epidemiology Collaboration (CKD-EPI) formula (13). The albumin-adjusted serum calcium(CaA) was calculated using the formula: (CaA)(mmol/L) = measured calcium (mmol/L) + [40-measured ALB (g/L)] × 0.02 (14).

The resting electrocardiogram before treatment was recorded by 12-channel electrocardiogram machine (EDAN SE-1201, Shenzhen, China) and automatically analyzed by the equipment software SEMIP. RHR and corrected QT (QTc) interval were collected. The QTc was calculated using the Bazett formula: QTc=QT/RR^(1/2) (15). All patients wore Holter devices (MOBI BI9800, Shenzhen, China) for 24 hours, with a data acquisition speed of 4000 Hz. The ECG data were processed by the professional dynamic electrocardiogram analysis system (BI-ECGLAB, developed by MOBI) and manually reviewed by the dynamic electrocardiogram technicians at the Affiliated Hospital of Qingdao University.

### Grouping and follow-up

The standard deviation of sinus RR interval (SDNN) is a time-domain indicator used to assess heart rate variability (HRV). It measures the standard deviation of the NN intervals (the time intervals between successive R-wave peaks in the ECG) over a specified period, reflecting the overall variability in heart rate. According to the HRV criteria recommended by the European Society of Cardiology and the Working Group of the North American Society for Pacing and Electrophysiology, SDNN in 24-hour dynamic electrocardiogram was used to determine whether the patient ‘s HRV was abnormal (6).The enrolled patients were divided into declined HRV group (SDNN < 100 ms) and normal HRV group (100 ms ≤ SDNN ≤ 180 ms). Patients were followed up by viewing medical records or making telephone calls. The follow-up deadline is May 31, 2024. Overall survival (OS) is defined as the time from NSCLC diagnosis to the date of death from any cause or the last follow-up.

### Statistical analysis

All analyses were conducted using SPSS version 26.0 (IBM Corp., Armonk, NY, USA). The Kolmogorov-Smirnov test was used to assess the normality of the variables. Normally distributed continuous variables were expressed as means ± standard deviations and compared using Student’s t-test. Non-normally distributed continuous variables were expressed as median (quartile 1; quartile 3), and comparisons between groups were made using the Mann-Whitney U test. Categorical variables were compared using the chi-square test. Univariate and multivariate logistic regression analyses were performed to identify significant predictors of HRV decline. Receiver operating characteristic (ROC) curves were constructed to evaluate the diagnostic performance of identified predictors. Kaplan-Meier survival curve and log-rank test were used to assess the prognostic value of the diagnostic index. Cox regression analysis and forest plots were employed to perform subgroup prognostic analysis. Statistical significance was set at *p* < 0.05.

## Results

### Comparison of baseline characteristics between declined HRV group and normal HRV group

A total of 131 eligible patients with newly diagnosed NSCLC were enrolled. Males accounted for 76.3%, aged 27-79 years, with a median age of 63 years. The proportions of stage I, stage II, stage III, and stage IV were 6.1%, 8.4%, 53.4%, and 32.1%, respectively. There were 59 patients in the normal HRV group and 72 patients in the declined HRV group. Significant differences in RHR, ALB, LDH, serum sodium and IL-6 were observed between the two groups (*p* < 0.05) (**Table 1**). The RHR, LDH and IL-6 in the declined HRV group were higher than those in the HRV normal group, while the serum sodium and ALB were lower. There was no significant difference in gender, age, body mass index(BMI), smoking status, tumor stage, QTc, hemoglobin, HDL-C, LDL-C, eGFR, UA, serum potassium and CaA between the two groups (*p* > 0.05) (**Table 1**).

**Table 1:** Comparison of baseline characteristics between declined HRV group and normal HRV group.

Characteristics	Total (n=131)	Normal HRV (n=59)	Declined HRV (n=72)	*P*
Male, n(%)	100(76.3)	46(78.0)	54(75.0)	0.691
Age (years)	63(58, 69)	62(58, 69)	64(58, 69)	0.370
BMI (kg/m^2^)	24(21, 26)	24(22, 26)	23(20, 26)	0.296
Smoking, n(%)	46(35.1)	17(28.8)	29(40.3)	0.171
tumor stage, n(%)				
stage I	8(6.1)	5(8.5)	3(4.2)	0.696
stage II	11(8.4)	6(10.2)	5(6.9)	
stage III	70(53.4)	30(50.8)	40(55.6)	
stage IV	42(32.1)	18(30.5)	24(33.3)	
RHR (b.p.m.)	75(66, 89)	70(63, 79)	84(72, 97)	<0.001
QTc interval(ms)	411 ± 23	412 ± 23	411 ± 22	0.866
Hemoglobin (g/L)	121 ± 16	123 ± 16	119 ± 15	0.122
ALB(g/L)	40 ± 4	41 ± 4	39 ± 4	0.031
HDL-C(mmol/L)	1.29 ± 0.36	1.30 ± 0.35	1.28 ± 0.38	0.795
LDL-C (mmol/L)	2.75(2.15, 3.43)	2.75(2.02, 3.31)	2.83(2.16, 3.64)	0.504
LDH (U/L)	189(154, 230)	182(146, 210)	202(164, 258)	0.010
eGFR (ml/min/1.73m^2^)	92 ± 19	94 ± 19	91 ± 20	0.259
UA (μmol/L)	281(246, 361)	299(257, 361)	279(227, 360)	0.449
potassium (mmol/L)	4.28(4.03, 4.50)	4.30(4.00, 4.52)	4.26(4.06, 4.46)	0.837
sodium(mmol/L)	137.5(135.5, 138.8)	138.7(137.4, 140.4)	136.3(134.6, 137.7)	<0.001
CaA (mmol/L)	2.33 ± 0.11	2.32 ± 0.10	2.33 ± 0.11	0.387
IL-6 (pg/ml)	8.45(4.53, 17.46)	5.67(4.53, 9.24)	11.42(7.69, 22.86)	<0.001

HRV, heart rate variability; BMI, body mass index; RHR, resting heart rate; QTc interval, corrected QT interval; ALB, albumin; HDL, high-density lipoprotein cholesterol, LDL, low-density lipoprotein cholesterol; LDH, lactate dehydrogenase; eGFR, estimated glomerular filtration rate; UA, uric acid; CaA, albumin-adjust serum calcium; IL-6, interleukin-6.

### Univariate logistic regression analysis and ROC analysis of clinical parameters

Univariate logistic regression analysis was performed on the enrolled clinical parameters. The result showed that RHR, ALB, LDH, serum sodium and IL-6 were significantly associated with the decline of HRV in newly diagnosed NSCLC patients (*p* < 0.05) (**Table 2**). Further ROC analysis was performed on the above five parameters to determine the cut-off value and diagnostic accuracy. The cut-off values were RHR > 82 b.p.m., ALB ≤ 38g/L, LDH > 197U/L, serum sodium ≤ 138.2mmol/L and IL-6 > 6.74pg/ml. The area under the curve (AUC) values of RHR, serum sodium and IL-6 were > 0.7, which had high diagnostic values (**Table 2**).

**Table 2:** Univariate logistic regression analysis and ROC analysis of clinical parameters.

	*P* (Univariate Logistic Regression)	AUC	Cut-off value	Sensitivity(%)	Specificity (%)
RHR	<0.001	0.767	>82 b.p.m.	59.72	84.75
ALB	0.033	0.607	≤38g/L	43.06	76.27
LDH	0.015	0.632	>197U/L	54.17	69.49
sodium	<0.001	0.795	≤138.2mmol/L	86.11	64.43
IL-6	0.002	0.764	>6.74pg/ml	84.72	64.41

RHR, resting heart rate; ALB, albumin; LDH, lactate dehydrogenase; IL-6, interleukin-6; AUC, area under the curve.

### Multivariate logistic regression analysis of clinical parameters and the development of diagnostic index

**Figure 1:**
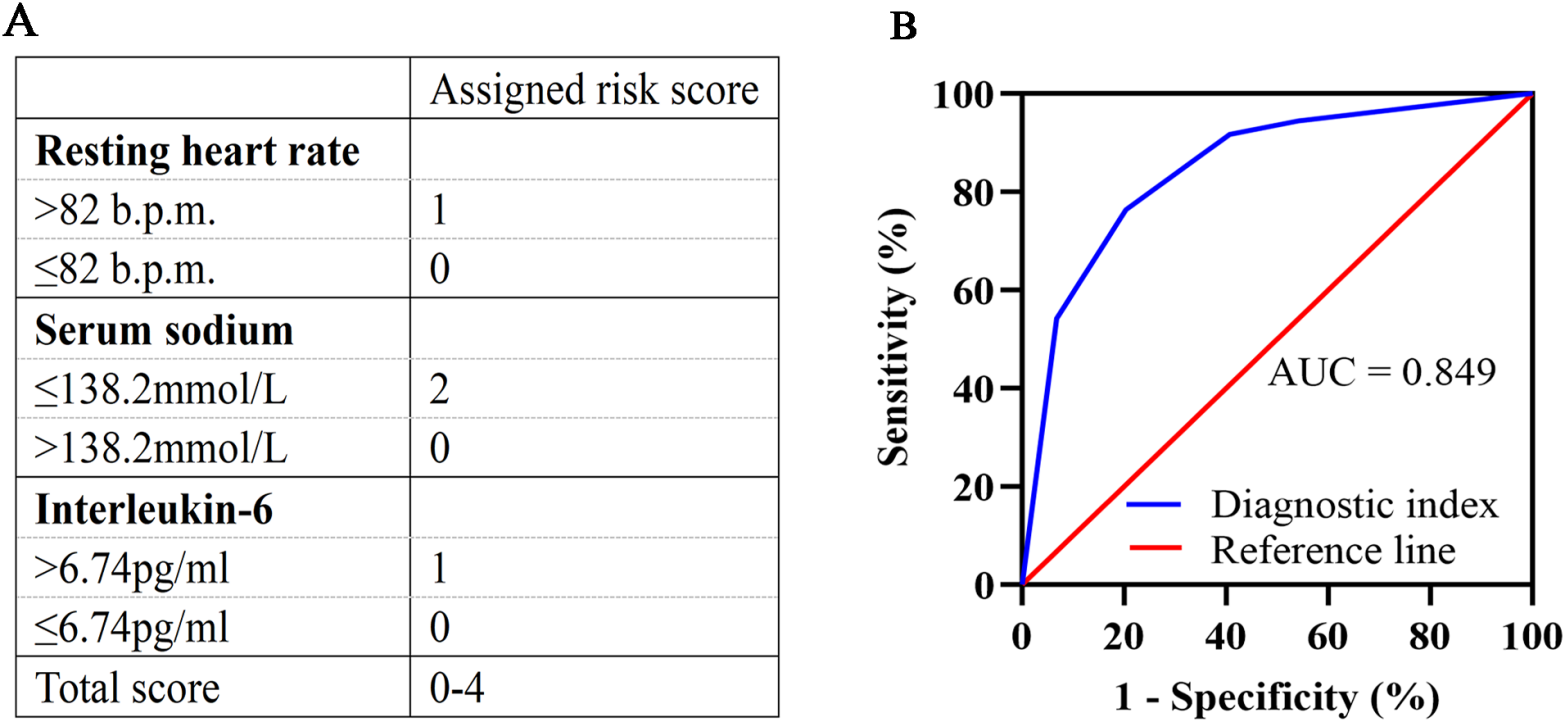
Development and validation of the diagnostic index. **(A)** Assigned risk scores for RHR, serum sodium, and IL-6. RHR >82 b.p.m. is assigned a score of 1, and ≤82 b.p.m. is assigned a score of 0. Serum sodium ≤138.2 mmol/L is assigned a score of 2, and >138.2 mmol/L is assigned a score of 0. IL-6 >6.74 pg/ml is assigned a score of 1, and ≤6.74 pg/ml is assigned a score of 0. Total scores range from 0 to 4. **(B)** ROC curve for the diagnostic index. The diagnostic index > 2 had the best predictive effect, with an AUC value of 0.849, a sensitivity of 76.39%, and a specificity of 79.66%. RHR, resting heart rate; IL-6, interleukin-6; ROC, receiver operating characteristic; AUC, area under the curve.

In multivariate logistic regression model analysis, RHR > 82 b.p.m [OR (95% CI), 3.143 (1.092-9.045), *p* = 0.034], serum sodium ≤ 138.2mmol/L [OR (95% CI), 6.806 (2.488-18.617), *p* < 0.001] and IL-6 > 6.74pg/ml [OR (95% CI), 3.203 (1.101-9.320), *p* = 0.033] were independent predictors of declined HRV in newly diagnosed NSCLC patients (**Table 3**). According to the regression coefficient and odds ratio, the assigned risk scores of RHR > 82 b.p.m.and IL-6 > 6.74 pg/ml were both 1, while the assigned risk score of serum sodium ≤ 138.2 mmol/L was 2 (**Figure 1A**). The diagnostic index for HRV in newly diagnosed NSCLC resulted in total risk scores between 0-4. ROC analysis showed that the diagnostic index > 2 had the best predictive effect, with an AUC value of 0.849, a sensitivity of 76.39%, and a specificity of 79.66% (**Figure 1B**).

**Table 3:** Multivariate logistic regression analysis of clinical parameters.

	β	S.E.	Wald	*P*	OR	95%CI
RHR	1.145	0.539	4.509	0.034	3.143	1.092~9.045
ALB	0.985	0.504	3.811	0.051	2.677	0.996~7.197
LDH	0.873	0.478	3.337	0.068	2.395	0.938~6.112
sodium	1.918	0.513	13.951	<0.001	6.806	2.488~18.617
IL-6	1.164	0.545	4.564	0.033	3.203	1.101~9.320

OR, odds ratio; CI, confidence interval; RHR, resting heart rate; ALB, albumin; LDH, lactate dehydrogenase; IL-6, interleukin-6.

### External validation of the diagnostic index

A total of 43 newly diagnosed NSCLC patients who met the inclusion criteria were included in the external validation cohort, with a comparison of the diagnostic index-related variable data provided in **Supplementary Table S1**. ROC analysis of the external validation data demonstrated an AUC of 0.788, with the best predictive performance observed when the diagnostic index was greater than 2, yielding a sensitivity of 83.33% and a specificity of 68.42% (**Supplementary Figure S1**). These results suggest that the diagnostic index possesses strong discriminative ability and effectively predicts the decline in HRV in patients with newly diagnosed NSCLC.

### Kaplan-Meier survival analysis based on HRV and diagnostic index

**Figure 2:**
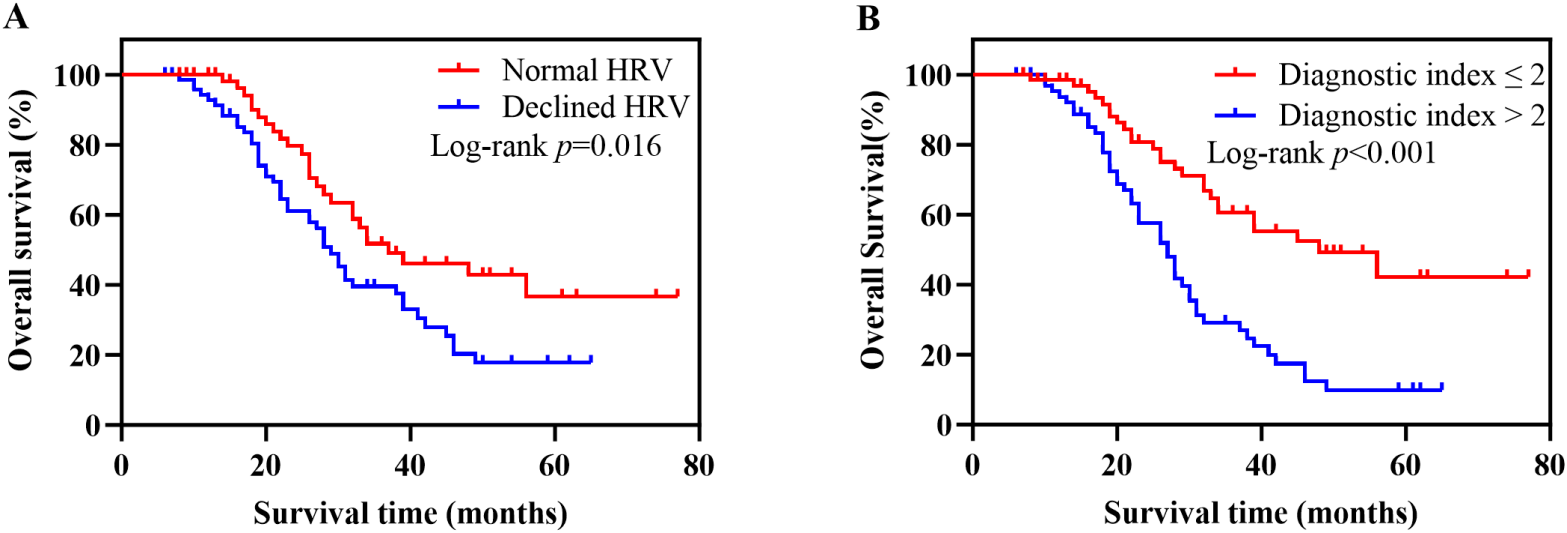
Kaplan-Meier survival analysis based on HRV and diagnostic index. **(A)** Kaplan-Meier survival curves for OS in patients with normal HRV versus declined HRV. Patients with declined HRV had significantly worse survival outcomes (log-rank p = 0.016). **(B)** Kaplan-Meier survival curves for OS stratified by the diagnostic index. Patients with a diagnostic index >2 exhibited significantly poorer OS compared to those with a diagnostic index ≤2 (log-rank *p* < 0.001). HRV, heart rate variability; OS, overall survival.

**Figure 3:**
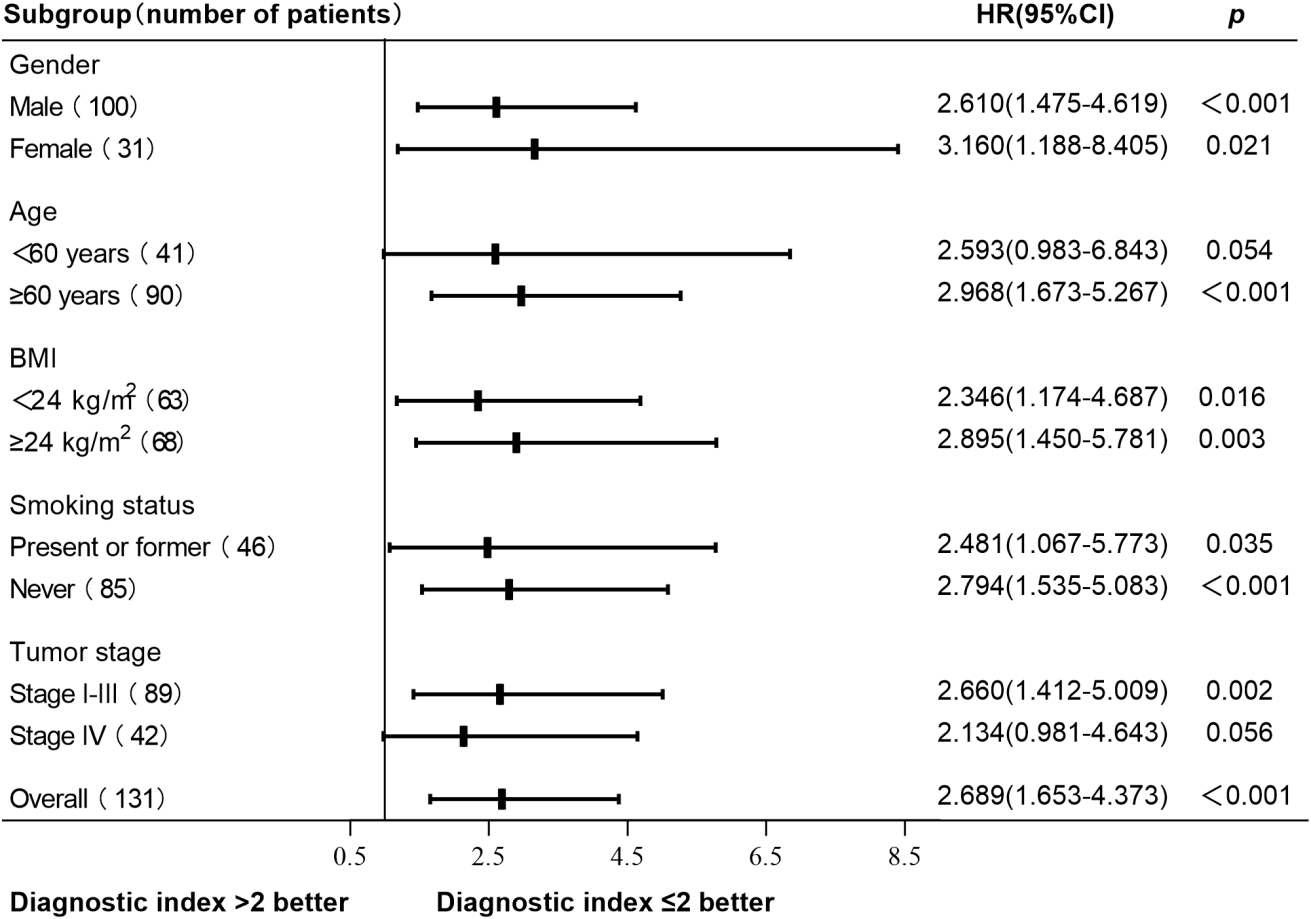
Subgroup analysis of OS based on the diagnostic index. Patients with diagnostic index ≤ 2 demonstrated better OS than patients with diagnostic index > 2 in all subgroup analyses. No statistically significant difference in OS was observed between patients with a diagnostic index ≤ 2 and those with a diagnostic index >2 in aged <60 years subgroup and stage IV subgroup (*p* values of 0.054 and 0.056, respectively). Other subgroups had statistical significance (*p* < 0.05). OS, overall survival; HR, hazard ratio; CI, confidence interval.

A total of 131 patients with newly diagnosed NSCLC were followed up until May 31, 2024. The median follow-up time was 26 months, 11 patients were lost to follow-up, and 72 patients died. The survival curve was constructed using Kaplan-Meier, and the log-rank test was used for comparison between groups. The median OS was 38 months (95% CI, 21-53) in the normal HRV group and 29 months (95% CI, 26-32) in the declined HRV group. There was a significant difference between the two groups (*p* = 0.016) (**Figure 2A**). According to the diagnostic index > 2, the patients were divided into 2 groups. Survival analysis showed that the median OS was 48 months (95% CI, 29-67) in the diagnostic index ≤ 2 group and 27 months (95% CI, 23-31) in the diagnostic index > 2 group, with significant difference (*p* < 0.001) (**Figure 2B**). Patients with diagnostic index ≤ 2 demonstrated better OS than patients with diagnostic index > 2 in all subgroup analyses. The difference in OS between the two groups was not statistically significant in the subgroups of patients aged <60 years and those with stage IV (*p* values of 0.054 and 0.056, respectively). Hazard ratio (HR) and two-sided 95% CI for each subgroup were listed in **Figure 3**.

## Discussion

This study developed a novel diagnostic index based on RHR, serum sodium, and IL-6 levels to predict HRV decline in newly diagnosed NSCLC patients. The index demonstrated significant prognostic value, with an AUC of 0.849, effectively stratifying patients by HRV status. Patients with a diagnostic index greater than 2 had significantly poorer OS compared to those with an index of 2 or less (log-rank *p* < 0.001). The subgroup analysis results showed that patients with a diagnostic index ≤ 2 exhibited better overall survival (OS) compared to those with a diagnostic index > 2 in all subgroups. However, the difference was not statistically significant in the subgroups of patients aged <60 years and those with stage IV disease (*p* values of 0.054 and 0.056, respectively), which may be attributable to the relatively small sample sizes, as well as the potential impact of age and disease stage on HRV (16). These findings highlight the prognostic value of HRV and its associated clinical parameters in NSCLC, suggesting that this diagnostic index can serve as a valuable tool for early identification and management of high-risk patients.

Our findings align with previous research indicating the prognostic value of HRV in various cancers. As early as 1991, J G van Dijk et al. (17) have proposed that RHR is associated with autonomic nervous function. Similar to Anker et al. (18), who identified increased RHR as a significant prognostic factor in treatment-naïve cancer patients, our study found that higher RHR is significantly associated with declined HRV and poorer outcomes in newly diagnosed NSCLC patients. Additionally, our study supports the results of Wu et al. (19), who demonstrated that low HRV predicts poor OS in lung cancer patients with brain metastases. These studies emphasize the critical role of autonomic dysfunction in cancer prognosis.However, our research differs in focusing on newly diagnosed NSCLC patients and incorporating a composite diagnostic index involving RHR, serum sodium, and IL-6 levels. This approach not only confirmed the prognostic value of RHR but also highlighted the significant contributions of serum sodium and IL-6, which were not as extensively explored in prior studies.

Serum sodium is a critical indicator of fluid balance and cellular function, with hyponatremia often correlating with poorer prognosis in cancer patients (20). Catalano M et al. (21) conducted a retrospective cohort study of patients with metastatic renal cell carcinoma who received immunotherapy. The results showed that compared with patients with serum sodium < 140 mmol/L, patients with serum sodium ≥ 140 mmol/L before and/or after immunotherapy had better OS and progression-free survival and higher disease control rate. Our study’s inclusion of serum sodium levels as a significant predictor aligns with Catalano. Moreover, previous studies have highlighted the role of IL-6 in cancer prognosis. Inoue et al. (22) identified IL-6 as a significant prognostic marker in NSCLC patients undergoing immunotherapy, supporting our inclusion of IL-6 as an independent predictor of HRV decline. Similarly, Liu et al. (23) found that IL-6 promotes NSCLC metastasis, reinforcing its importance in our index. These findings are consistent with our results and underscore the multifaceted role of IL-6 in NSCLC prognosis. In our research, the combined use of RHR, serum sodium, and IL-6 enhances the predictive accuracy and clinical relevance of the diagnostic index, offering a robust method for early identification of high-risk patients.

The diagnostic index developed in this study has significant potential to impact clinical practice. By enabling early identification of HRV decline, this tool can inform treatment decisions and patient management strategies. For instance, patients identified as high-risk based on their diagnostic index could receive more intensive monitoring and tailored therapeutic interventions. Studies have shown that ivabradine can improve the autonomic nervous function of lymphoma patients after radiotherapy by reducing RHR and improving HRV, without affecting the exercise ability and quality of life of patients (24). In recent years, *in vitro* studies have shown that due to the imbalance of intracellular signaling pathways, cells grown under low sodium conditions have higher proliferation and motility. Antidiuretic hormone receptor antagonists can effectively inhibit the proliferation and invasion of cancer cells, which may open up a new situation in the pharmacological strategy for the treatment of cancer (25, 26).

This personalized approach could improve patient outcomes by addressing the specific physiological and pathological conditions indicated by their HRV status. Furthermore, integrating HRV assessment into routine clinical evaluations could enhance the overall care quality for NSCLC patients. The ability to predict HRV decline and its associated poor outcomes can facilitate timely and tailored interventions, potentially improving patient management and survival rates. The incorporation of IL-6 into the diagnostic index highlights the importance of inflammatory processes in cancer prognosis. Alteration in T cell populations and function may be a mechanism underlying the poor prognosis seen in NSCLC patients with high IL-6 levels (27). Our study corroborates these findings, demonstrating that IL-6 is a significant predictor of HRV decline and OS in NSCLC patients. IL-6, a pro-inflammatory cytokine, has a broad impact on tumorigenesis, growth and progression, with its signaling pathway playing a key role in the invasion and metastatic formation of cancer cells (28, 29). In addition, studies have shown that IL-6 regulates GABAA receptors in the dorsomedial hypothalamic nucleus (DMH) to affect HRV through activation of the JAK/STAT pathway, while vagal nerve activity modulates the inflammatory response by reducing cytokine release (30, 31). The inclusion of IL-6 in our diagnostic index enhances its predictive accuracy and underscores the importance of inflammatory processes in cancer prognosis.

Despite the promising findings, this study has some limitations. Firstly, the retrospective design may introduce selection bias and limit causal inference. HRV is influenced by a variety of factors, including pathological, physiological, psychological, environmental, lifestyle and genetic factors (16). To ensure the reliability of the study results, potential confounders that could affect HRV, such as heart disease, diabetes mellitus, mental illness and the use of medications affecting heart rate, were systematically excluded during the study design. Furthermore, subgroup analyses based on factors such as gender, age, BMI, smoking and cancer stage were conducted. However, excluding these HRV-related factors may limit the generalizability of the findings, and the relatively small sample size in the subgroup analyses may also impact the results. Secondly, baseline measurements may not capture the dynamic changes in HRV and related biomarkers over time. Future studies should adopt a prospective design with larger and more diverse populations, increase the sample size for subgroup analyses and external validation, and further investigate the temporal changes in HRV and biomarkers to enhance the generalizability and reliability of the conclusions. Additionally, exploring and integrating new biomarkers could enhance the index’s predictive accuracy and clinical relevance.

## Conclusions

This study introduces a diagnostic index based on RHR, serum sodium, and IL-6 levels to predict heart rate HRV decline in newly diagnosed NSCLC patients. The index shows significant prognostic value, aiding early identification and management of high-risk patients. Its implementation in clinical practice could enhance personalized treatment strategies, potentially improving patient outcomes. Future research should validate this index in larger, diverse populations and through prospective studies to increase its robustness. Longitudinal studies are essential to understand the dynamic changes in HRV and biomarkers over time. Additionally, integrating new biomarkers could further improve the index’s predictive accuracy and clinical relevance, contributing to better management and personalized therapeutic interventions for NSCLC patients.

## Data Availability

The raw data supporting the conclusions of this article will be made available by the authors, without undue reservation.

## Supplementary material

Supplementary Table S1 Index-relevant variable data for external validation. HRV, heart rate variability; RHR, resting heart rate; IL-6, interleukin-6.

Supplementary Figure S1 ROC curve for the external validation of the diagnostic index. The AUC was 0.788, with the diagnostic index demonstrating the best predictive performance when the value exceeded 2. At this threshold, the sensitivity was 83.33% and the specificity was 68.42%. ROC, receiver operating characteristic; AUC, area under the curve.
