# Corrigendum: A diagnostic index for predicting heart rate variability decline and prognostic value in newly diagnosed non-small cell lung cancer patients

**DOI:** 10.3389/fonc.2025.1566535

**Published:** 2025-02-12

**Authors:** Lifang Zhang, Ying Liu, Di Han, Yan Wang, Fanqi Geng, Wei Ding, Xuejuan Zhang

**Affiliations:** ^1^ Department of General Medicine, The Affiliated Hospital of Qingdao University, Qingdao, China; ^2^ Department of Infectious Disease, The Affiliated Hospital of Qingdao University, Qingdao, China

**Keywords:** heart rate variability, non-small cell lung cancer, diagnostic index, resting heart rate, serum sodium, interleukin-6, overall survival

In the published article, there was an error. The main text contains an inconsistency with the data presented in Table 1.

A correction has been made to **Results**, *Comparison of baseline characteristics between declined HRV group and normal HRV group*, Paragraph 1. This sentence previously stated:

“The proportions of stage I, stage II, stage III, and stage IV were 1.5%, 8.4%, 55.0%, and 35.1%, respectively.”

The corrected sentence appears below:

“The proportions of stage I, stage II, stage III, and stage IV were 6.1%, 8.4%, 53.4%, and 32.1%, respectively.”

The authors apologize for this error and state that this does not change the scientific conclusions of the article in any way. The original article has been updated.

