# Supplementary material for: A diagnostic index for predicting heart rate variability decline and prognostic value in newly diagnosed non-small cell lung cancer patients
Source: Front Oncol. 2024 Dec 4;14:1463805. doi: 10.3389/fonc.2024.1463805 (PMC11652349; doi:10.3389/fonc.2024.1463805)
Supplement: Supplementary Table S1 — Index-relevant variable data for external validation. HRV, heart rate variability; RHR, resting heart rate; IL-6, interleukin-6. [file Table1.docx]

**Supplemental Table 1** Index-relevant variable data for external validation

| Variable | Total  （n=43） | normal HRV  （n=19） | declined HRV  （n=24） | *p* |
| --- | --- | --- | --- | --- |
| RHR (b.p.m.) | 78（70，87） | 73（64，83） | 84（71，92） | 0.028 |
| sodium(mmol/L) | 136.5（135.3，138.1） | 137.7（136.1，139.0） | 136.2（135.0，137.5） | 0.032 |
| IL-6 (pg/ml) | 8.54（5.91，18.46） | 6.24（2.19，10.14） | 15.27（7.75，22.23） | 0.002 |

HRV, heart rate variability; RHR, resting heart rate; IL-6, interleukin-6.
