# Supplementary figures and images for: A diagnostic index for predicting heart rate variability decline and prognostic value in newly diagnosed non-small cell lung cancer patients
Source: Front Oncol. 2024 Dec 4;14:1463805. doi: 10.3389/fonc.2024.1463805 (PMC11652349; doi:10.3389/fonc.2024.1463805)

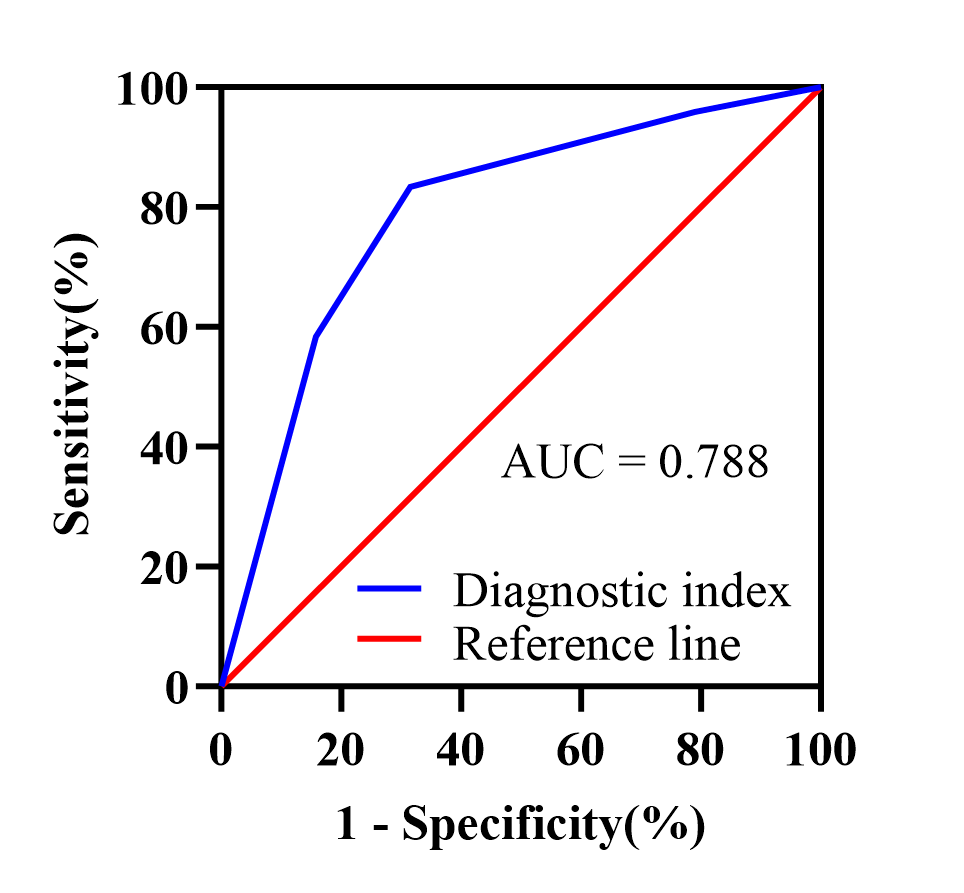

Supplement: Supplementary Figure S1 — ROC curve for the external validation of the diagnostic index. The AUC was 0.788, with the diagnostic index demonstrating the best predictive performance when the value exceeded 2. At this threshold, the sensitivity was 83.33% and the specificity was 68.42%. ROC, receiver operating characteristic; AUC, area under the curve. [file Image1.tif]
